# Supplementary material for: A Hybrid Modeling Framework for Predictive Digital Twins of CHO Cell Culture
Source: Comput Struct Biotechnol J. 2026 May 4;35(1):0078. doi: 10.34133/csbj.0078 (PMC13136614; doi:10.34133/csbj.0078)

**Supplementary Figures and Tables**

**A Hybrid Modeling Framework for Predictive Digital Twins of CHO Cell Culture**

**Supplementary Figure 1**. Relative metabolite concentration profiles over time for 23 fed-batch CHO-S cell cultures. The colors in the figure represent the eight distinct FMA+FMB formulations tested. For cultures with the same formulation, the volumes of FMA+FMB added varied, while the timing of additions was consistent across all experiments.


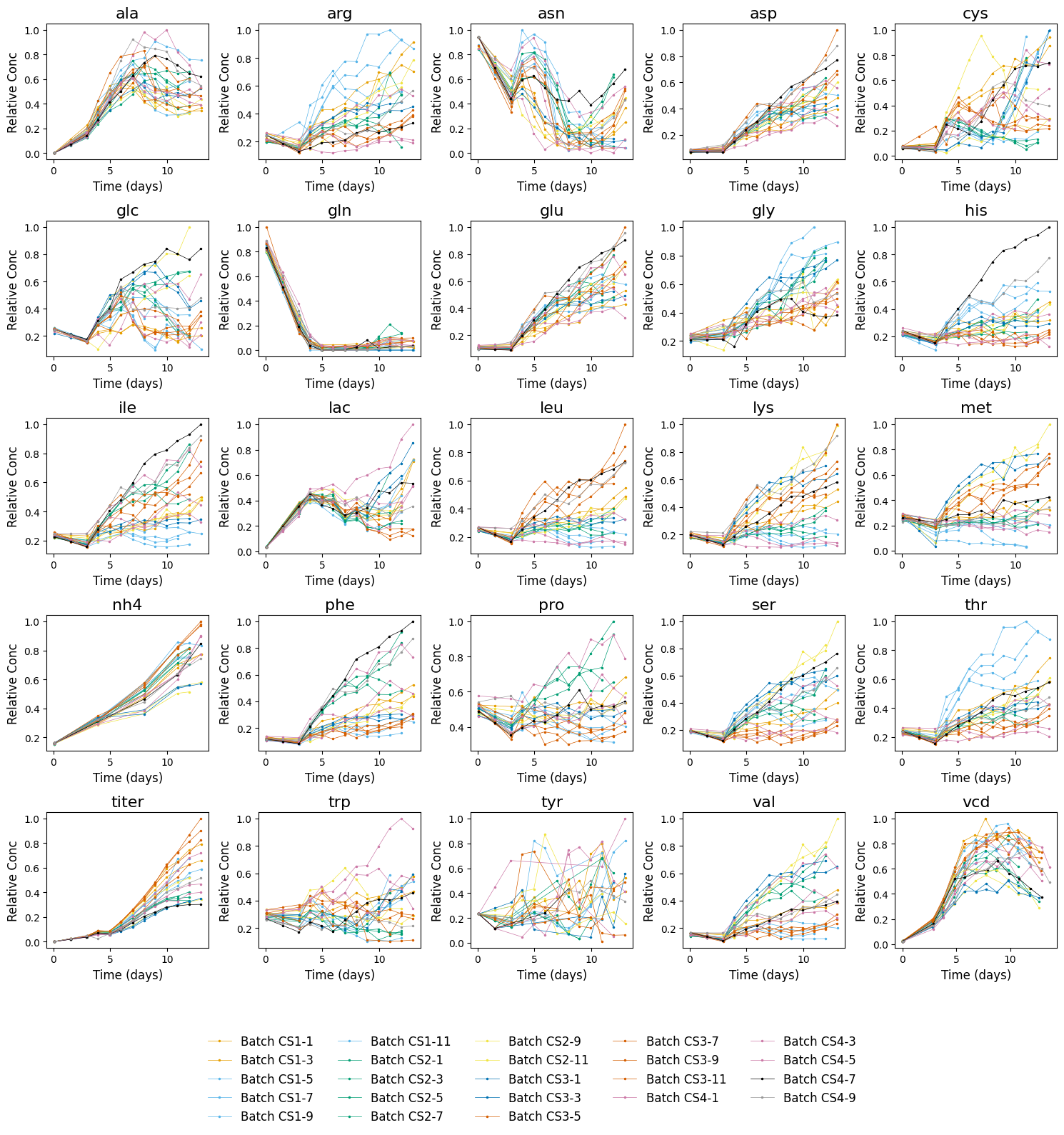


**Supplementary Figure 2**. Schematic representation of the recurrent neural network (RNN) architecture used for growth rate prediction.


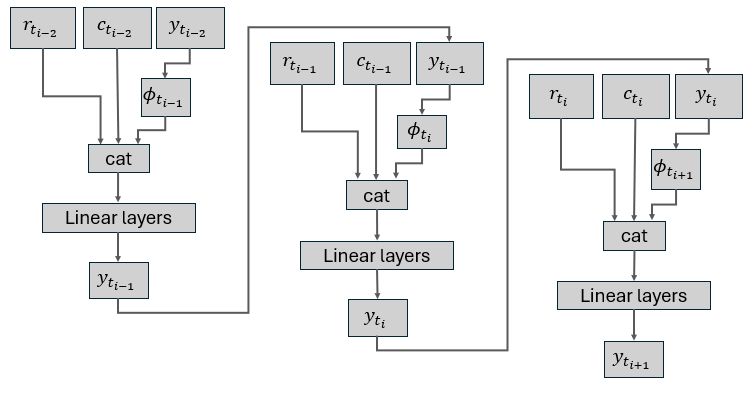


**Supplementary Figure 3.** Comparison between simulated and experimental viable cell density (VCD) trajectories obtained by integrating the NN-predicted growth rates within the ODE framework. The shaded regions denote the MetRac confidence intervals.


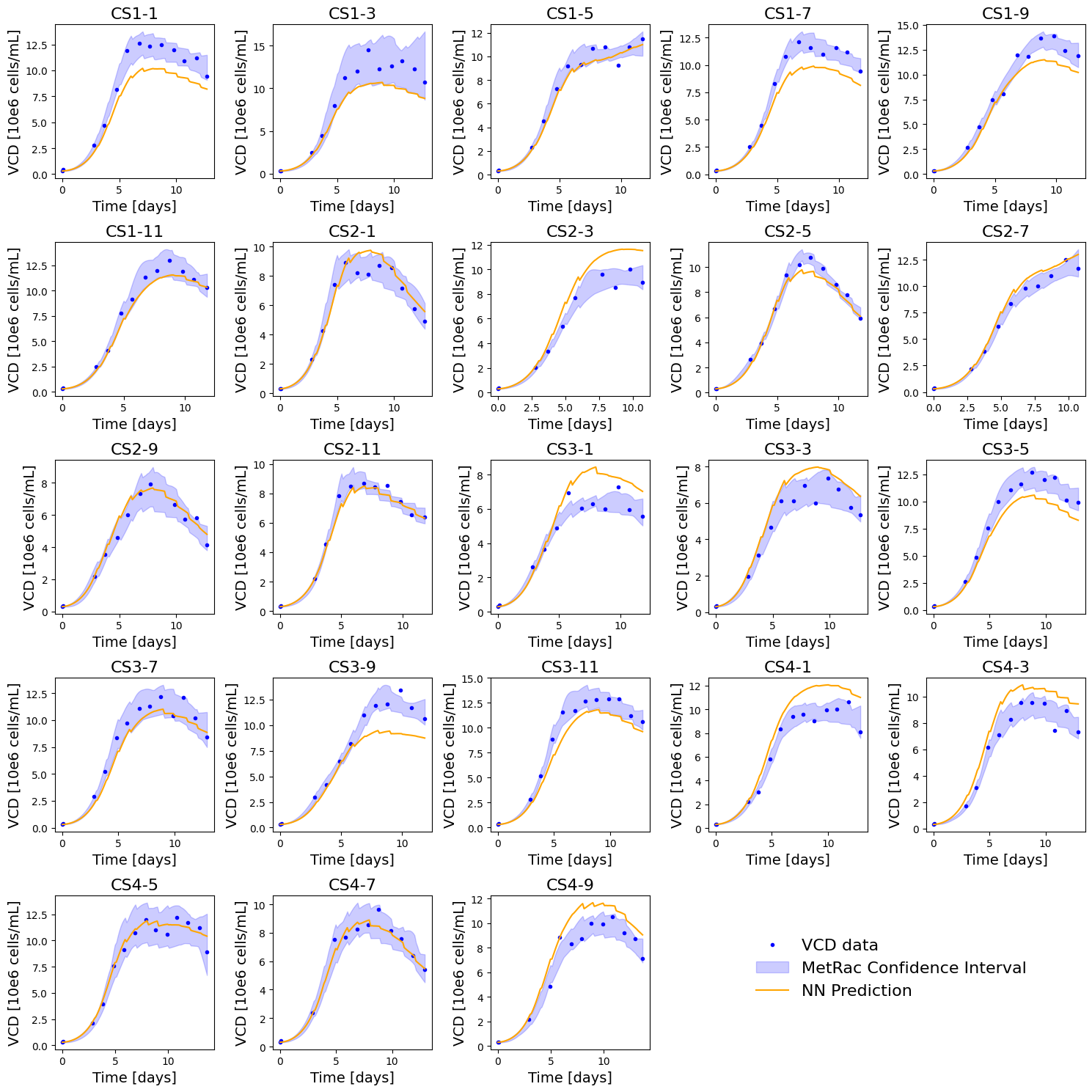


**Supplementary Figure 4.** Overview of model fits and simulated state variables for all batches. Each row corresponds to one batch. Columns 1 and 2 show the fitted model simulations (solid lines) and experimental data (points) for viable and dead cell concentrations, respectively. Columns 3 and 4 display the simulated trajectories of lysed cells and biomaterial (metabolic by-products), which are internal state variables not directly measured experimentally.


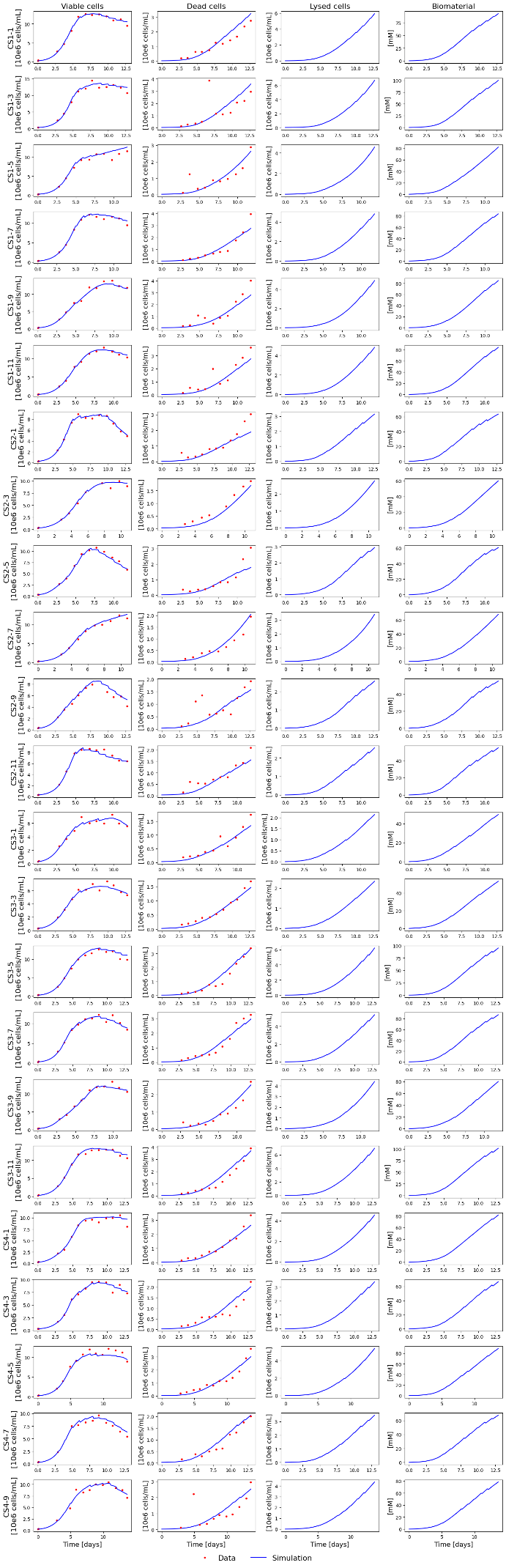


**Supplementary Figure 5.** Detailed metabolite time-course simulations for all batches using the FLEX ODE model. Comparison between experimental data (blue dots), simulation results (orange lines), and confidence intervals (shaded regions) for the five FLEX metabolites: glucose, lactate, glutamine, glutamate, and ammonia. The experimental and simulated concentration data for glucose, lactate, glutamine, glutamate, and ammonia are normalized to be displayed on a relative scale.

**
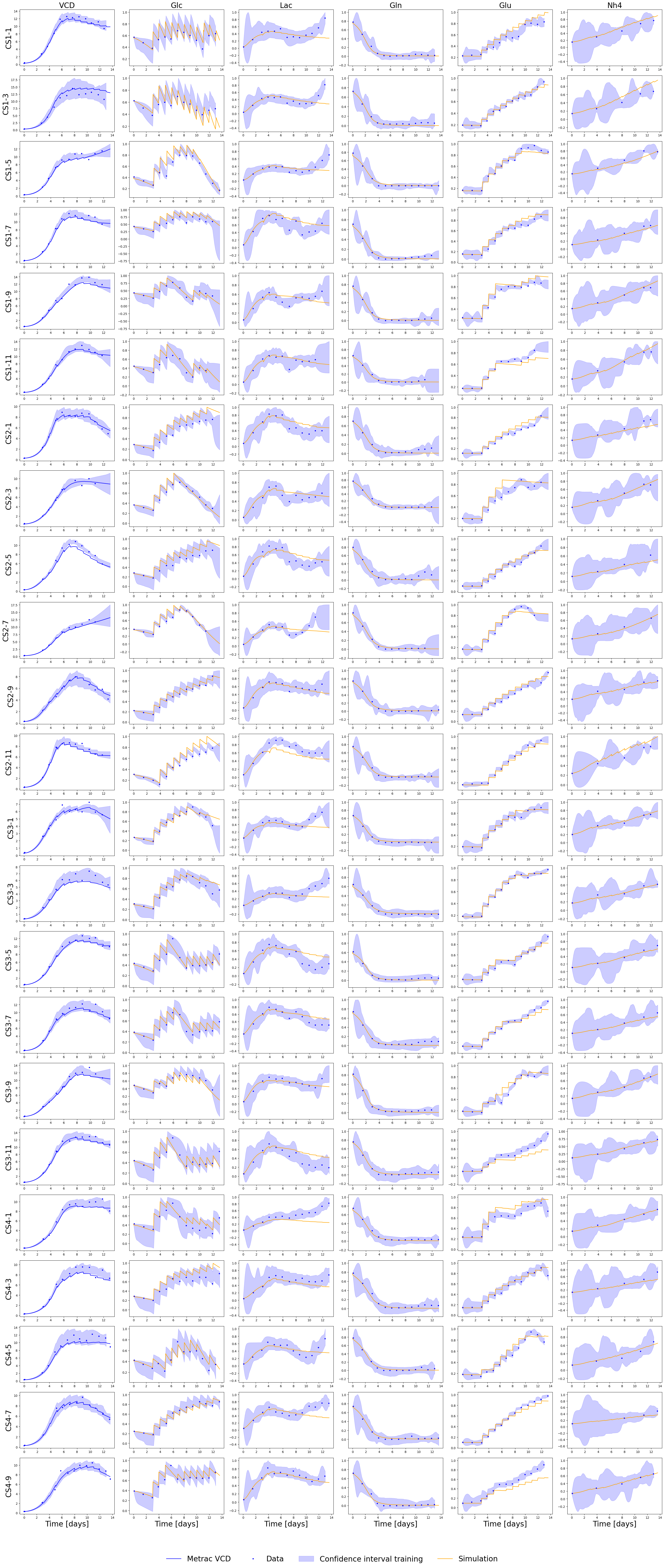
**

**Supplementary Figure 6.** Metabolite-wise prediction errors for PC-dFBA models. Top panel: Boxplots of log-transformed summed squared errors (log(SSE)) for each metabolite under the three validation strategies (direct, LOMO, LOBO). Bottom panel: Comparison of log(SSE) distributions between Model_Flex and Model_All across metabolites.


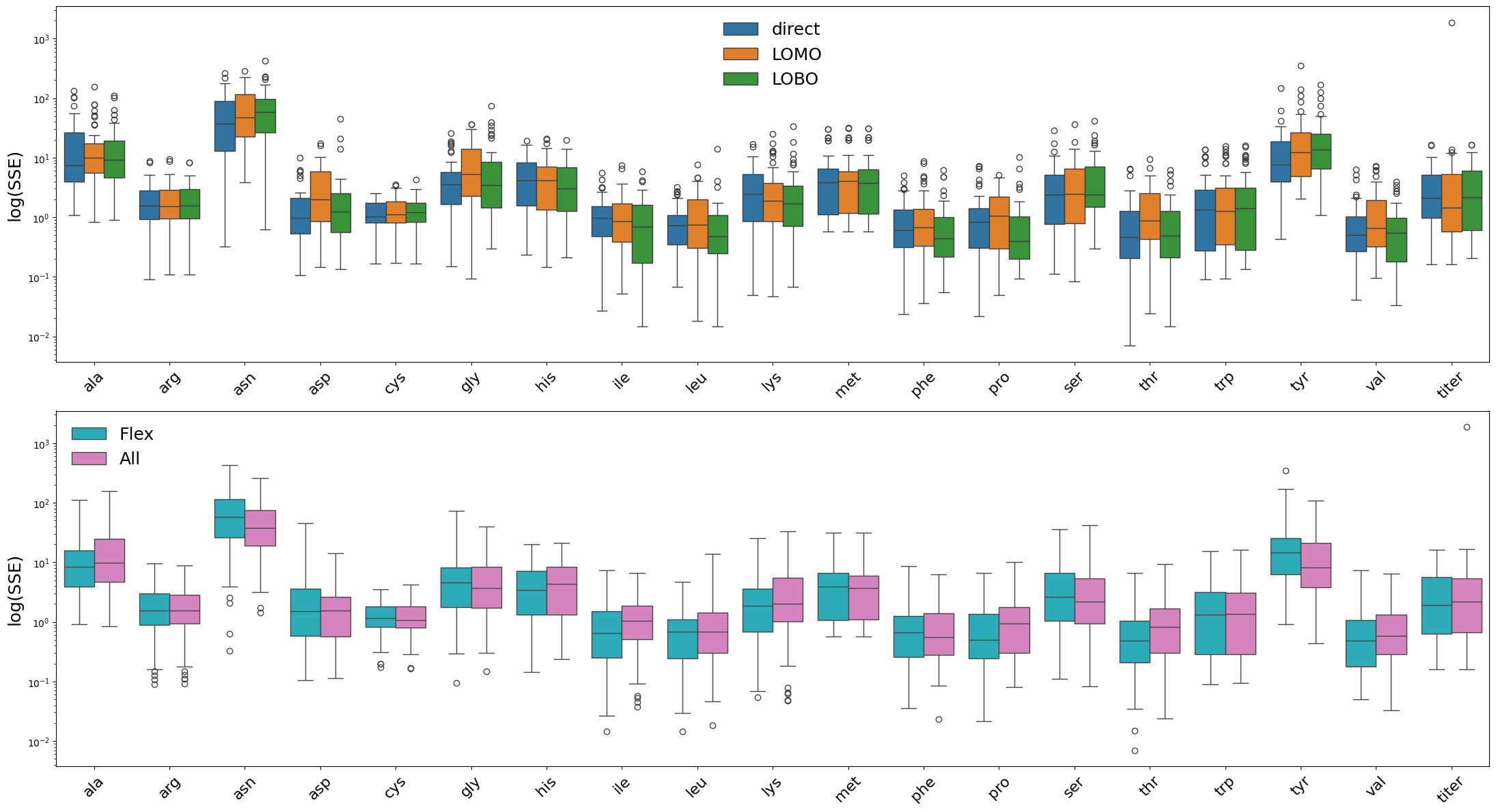


**Supplementary Figure 7.** PCA-dFBA prediction performance across validation strategies. (A) Coefficient of determination (R²) for 19 extracellular metabolites obtained using the original PCA-dFBA formulation across the three validation strategies (direct, LOMO, and LOBO). (B) Total log-transformed summed squared error (log(SSE)) for the same simulations.


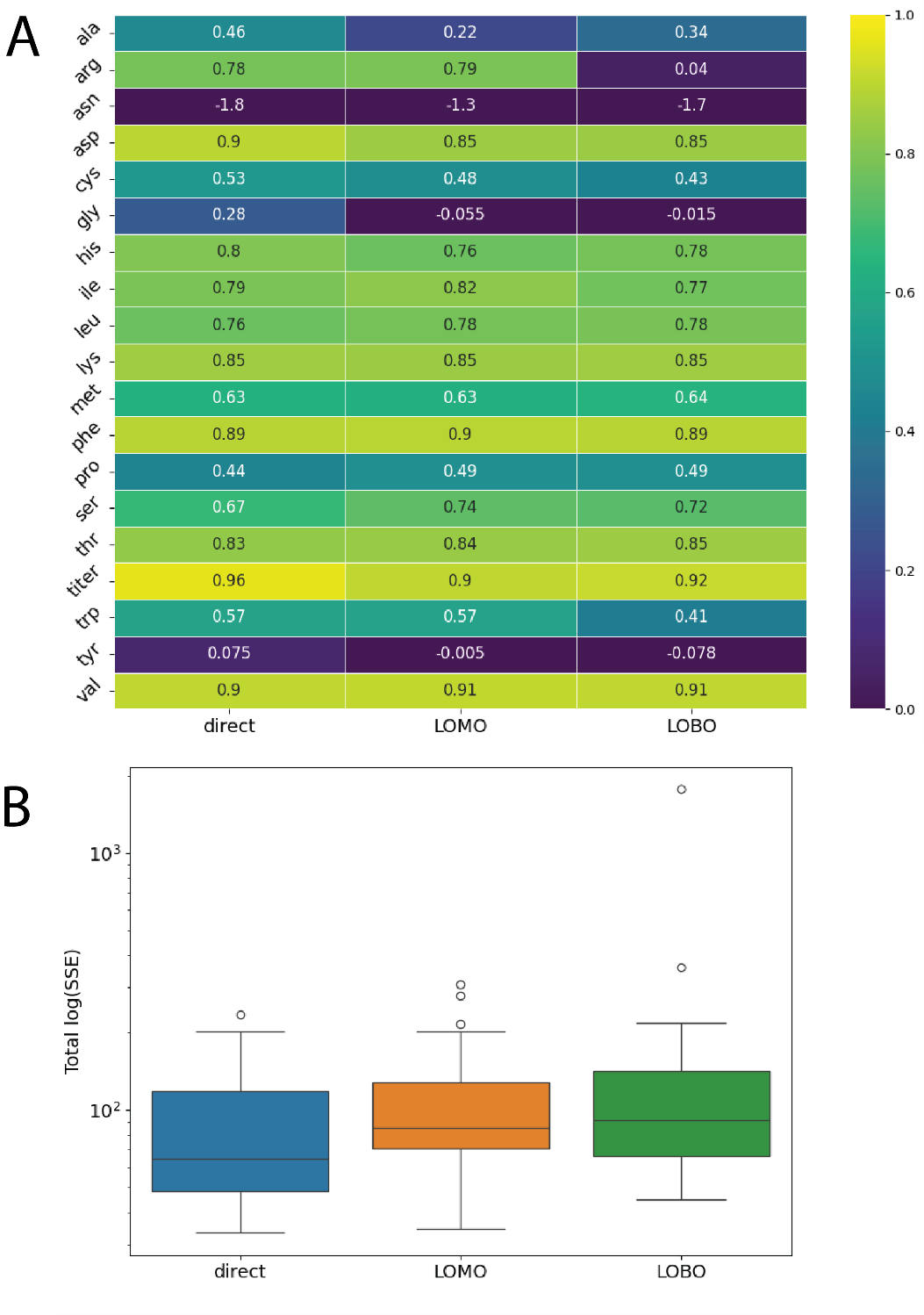


**Supplementary Figure 8.**  **Example of the propagation of the prediction variability of the PC-dFBA algorithm.** Comparison of predicted (orange) and experimental (blue) time courses for viable-cell density, product titer, and 23 extracellular metabolites for batch CS4-5 under the LOMO_Flex configuration. The shaded blue regions represent MetRaC-derived confidence intervals. The shaded green regions represent FVA simulation envelope.


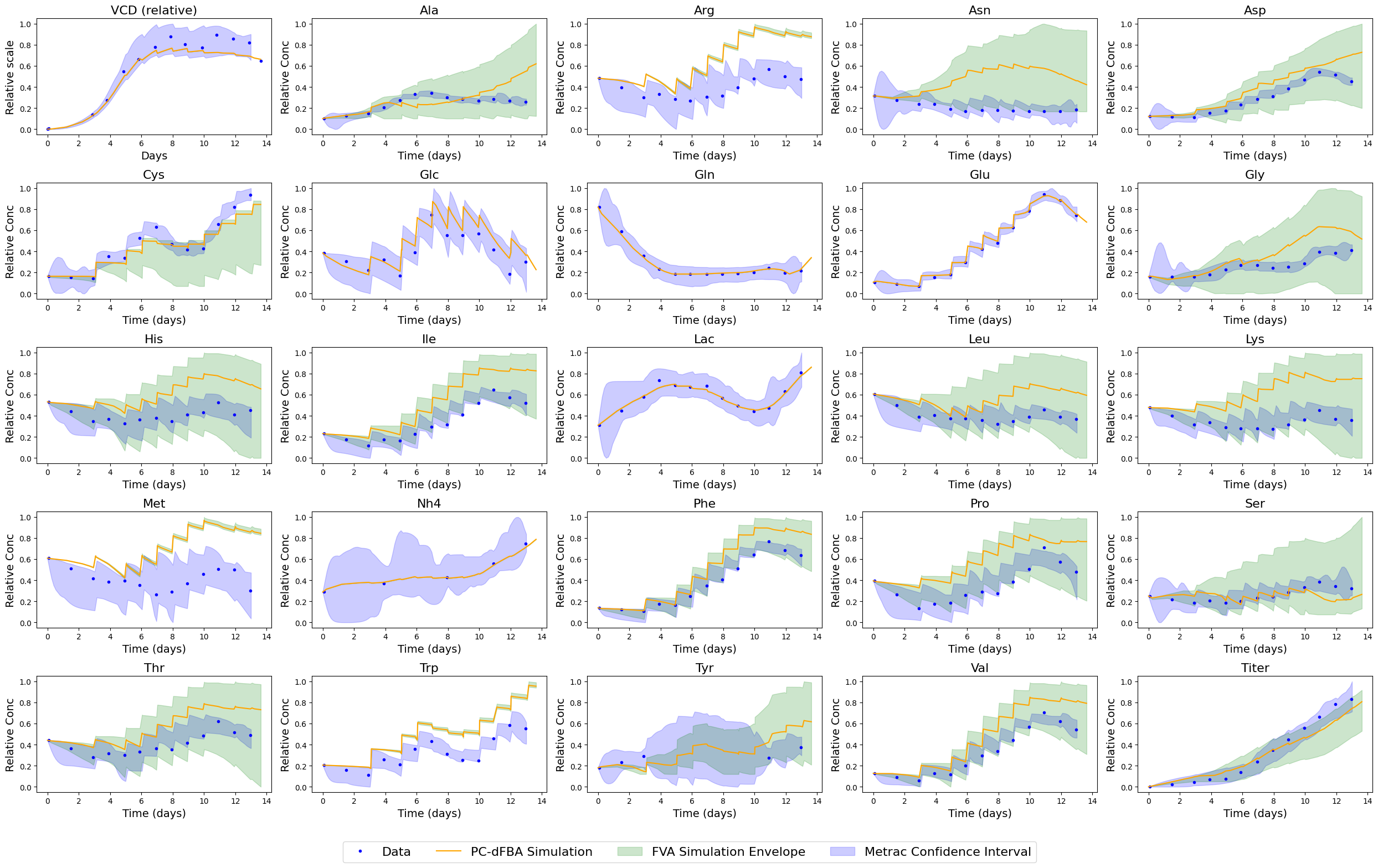

Supplement: Supplementary 1 — Methods Figs. S1 to S8 Tables S1 and S2 [file csbj.0078.f1.zip › Revised Supplementary Figures.docx]
