# Supplementary material for: A Hybrid Modeling Framework for Predictive Digital Twins of CHO Cell Culture
Source: Comput Struct Biotechnol J. 2026 May 4;35(1):0078. doi: 10.34133/csbj.0078 (PMC13136614; doi:10.34133/csbj.0078)
Supplement: Supplementary 1 — Methods Figs. S1 to S8 Tables S1 and S2 [file csbj.0078.f1.zip › Supplementary Figures & Tables.pdf]

Supplementary Figures and Tables

A Hybrid Modeling Framework for Predictive Digital Twins of CHO Cell Culture

Supplementary Figure 1. Relative metabolite concentration profiles over time for 23 fed-batch CHO-S cell cultures. The colors in the figure represent the eight distinct FMA+FMB formulations tested. For cultures with the same formulation, the volumes of FMA+FMB added varied, while the timing of additions was consistent across all experiments.

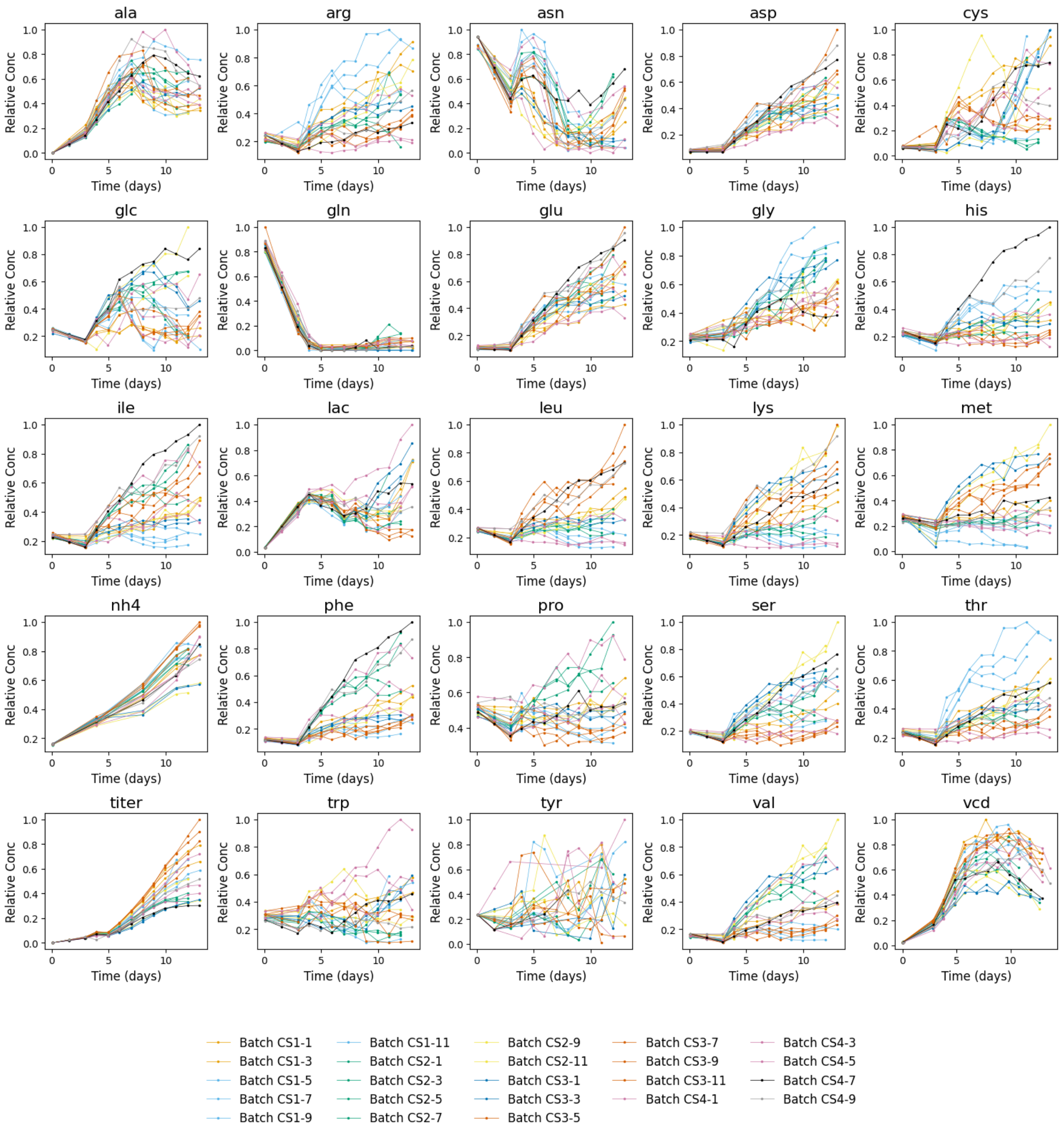

Supplementary Figure 2. Schematic representation of the recurrent neural network (RNN) architecture used for growth rate prediction.

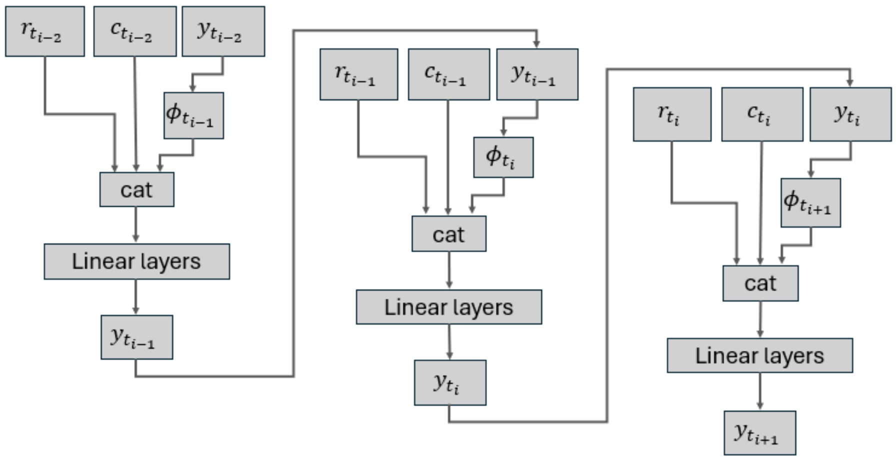

Supplementary Figure 3. Comparison between simulated and experimental viable cell density (VCD) trajectories obtained by integrating the NN-predicted growth rates within the ODE framework. The shaded regions denote the MetRac confidence intervals.

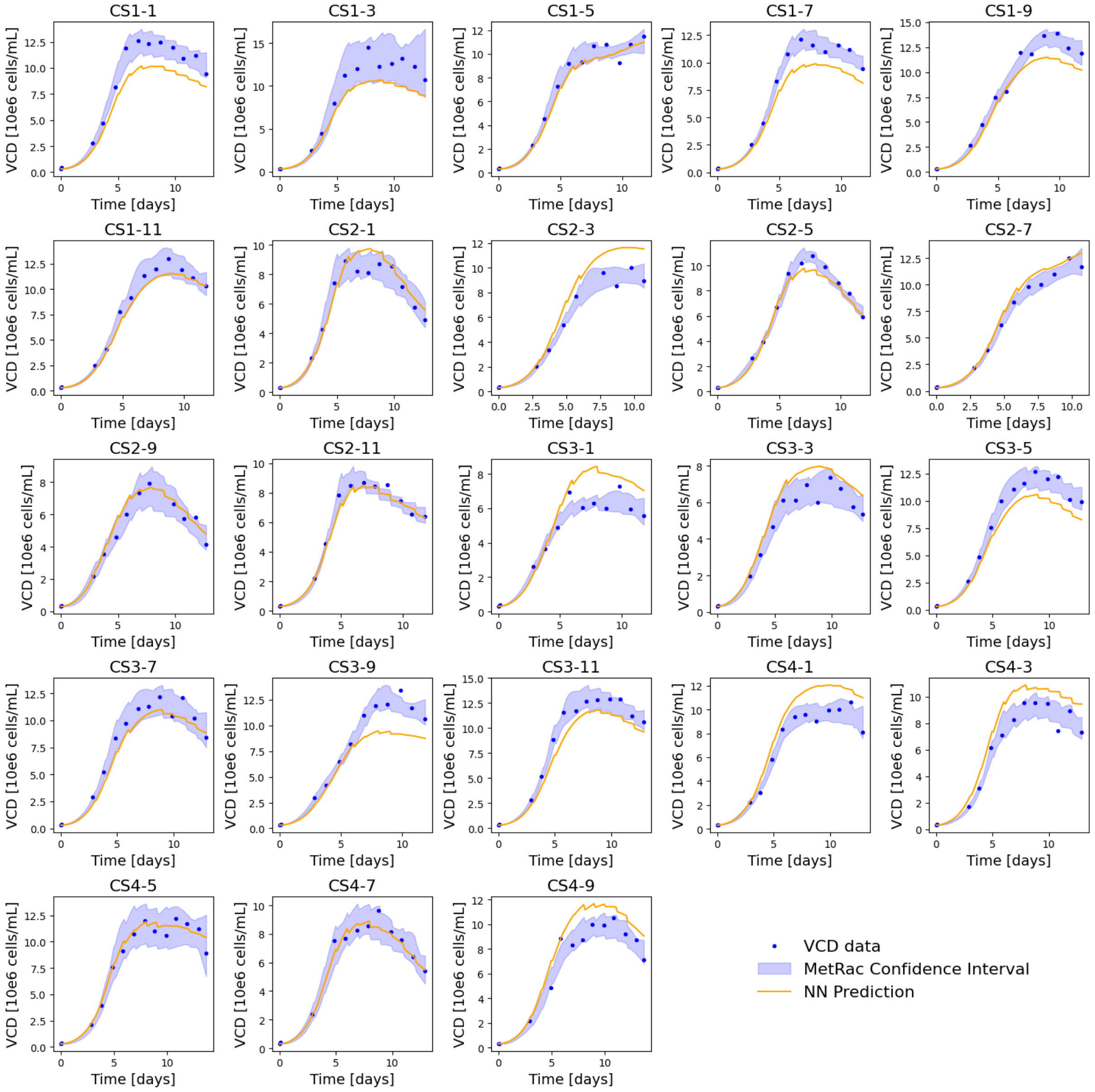

Supplementary Figure 4. Overview of model fits and simulated state variables for all batches. Each row corresponds to one batch. Columns 1 and 2 show the fitted model simulations (solid lines) and experimental data (points) for viable and dead cell concentrations, respectively. Columns 3 and 4 display the simulated trajectories of lysed cells and biomaterial (metabolic by-products), which are internal state variables not directly measured experimentally.

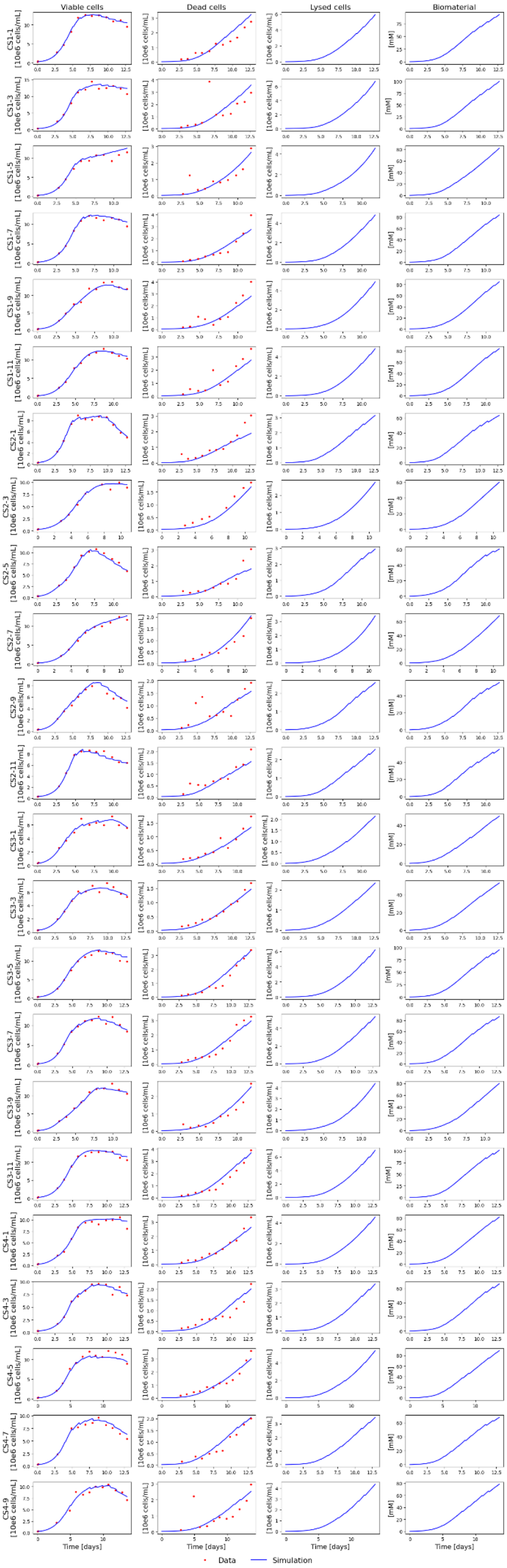

Supplementary Figure 5. Detailed metabolite time-course simulations for all batches using the FLEX ODE model. Comparison between experimental data (blue dots), simulation results (orange lines), and confidence intervals (shaded regions) for the five FLEX metabolites: glucose, lactate, glutamine, glutamate, and ammonia. The experimental and simulated concentration data for glucose, lactate, glutamine, glutamate, and ammonia are normalized to be displayed on a relative scale.

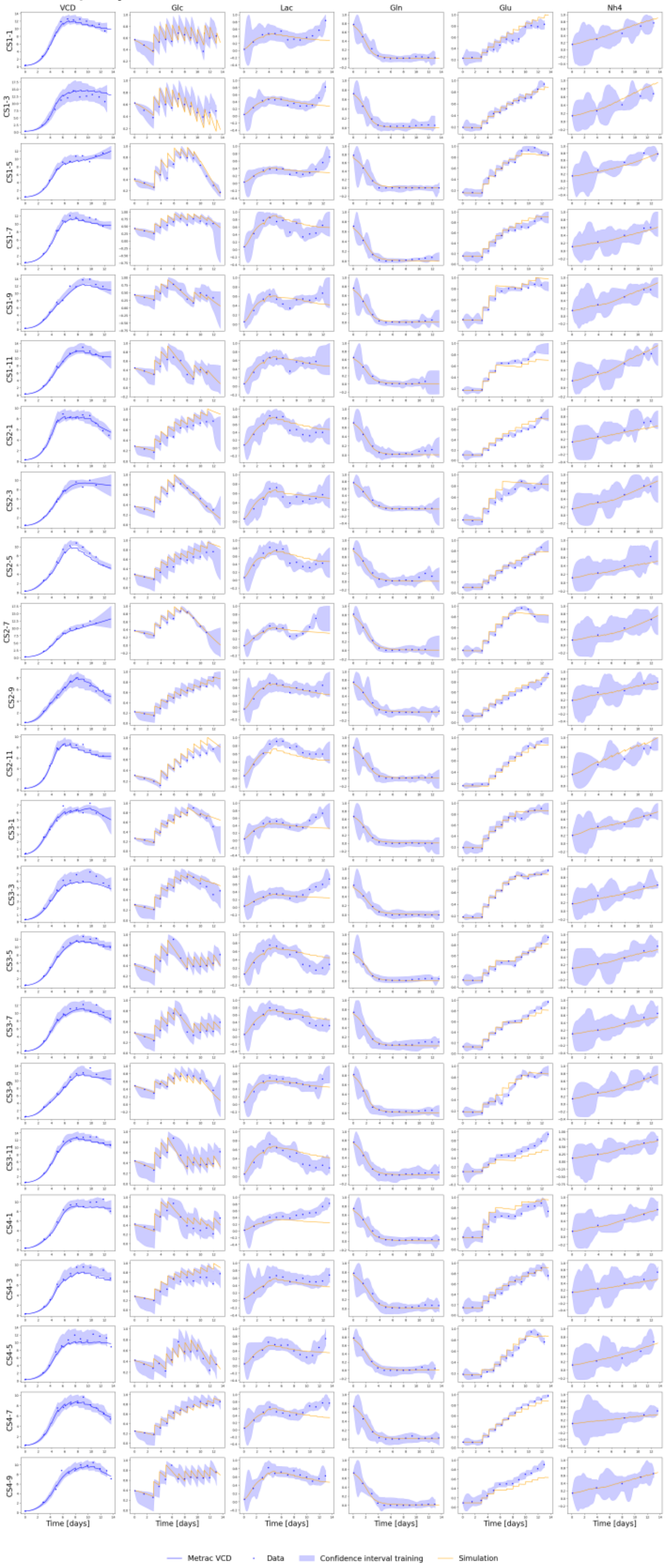

Supplementary Figure 6. Metabolite-wise prediction errors for PC-dFBA models. Top panel: Boxplots of log-transformed summed squared errors (log(SSE)) for each metabolite under the three validation strategies (direct, LOMO, LOBO). Bottom panel: Comparison of log(SSE) distributions between Model_Flex and Model_All across metabolites.

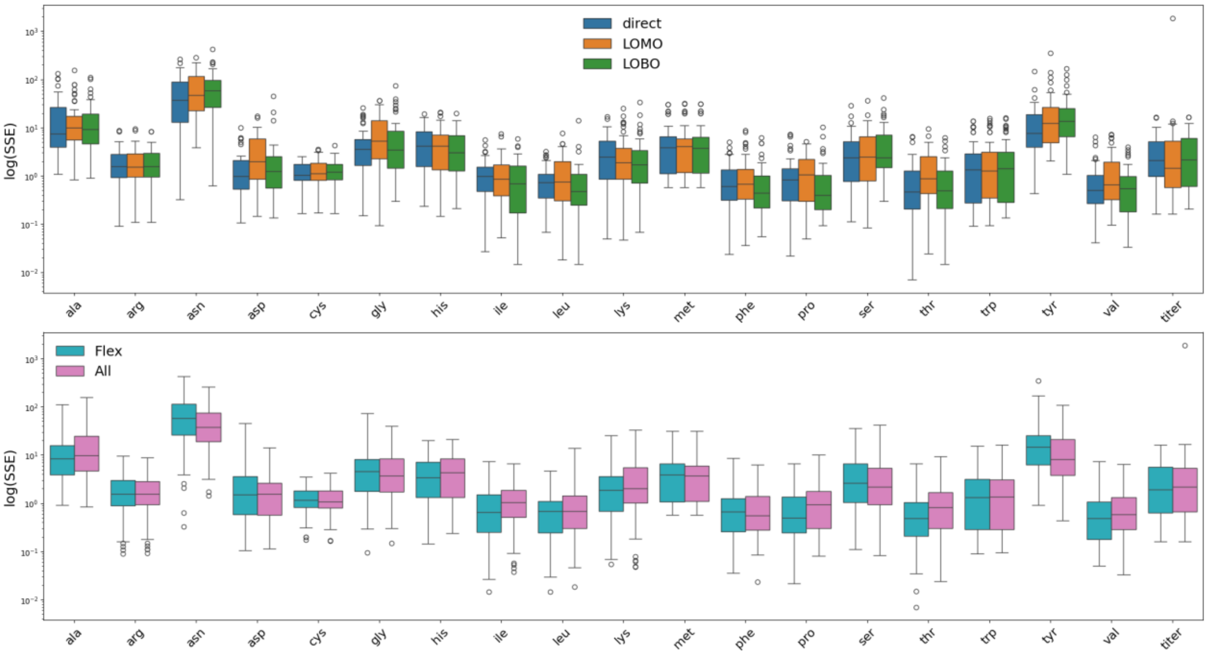

Supplementary Figure 7. PCA-dFBA prediction performance across validation strategies. (A) Coefficient of determination (R^2) for 19 extracellular metabolites obtained using the original PCA-dFBA formulation across the three validation strategies (direct, LOMO, and LOBO). (B) Total log-transformed summed squared error (log(SSE)) for the same simulations.

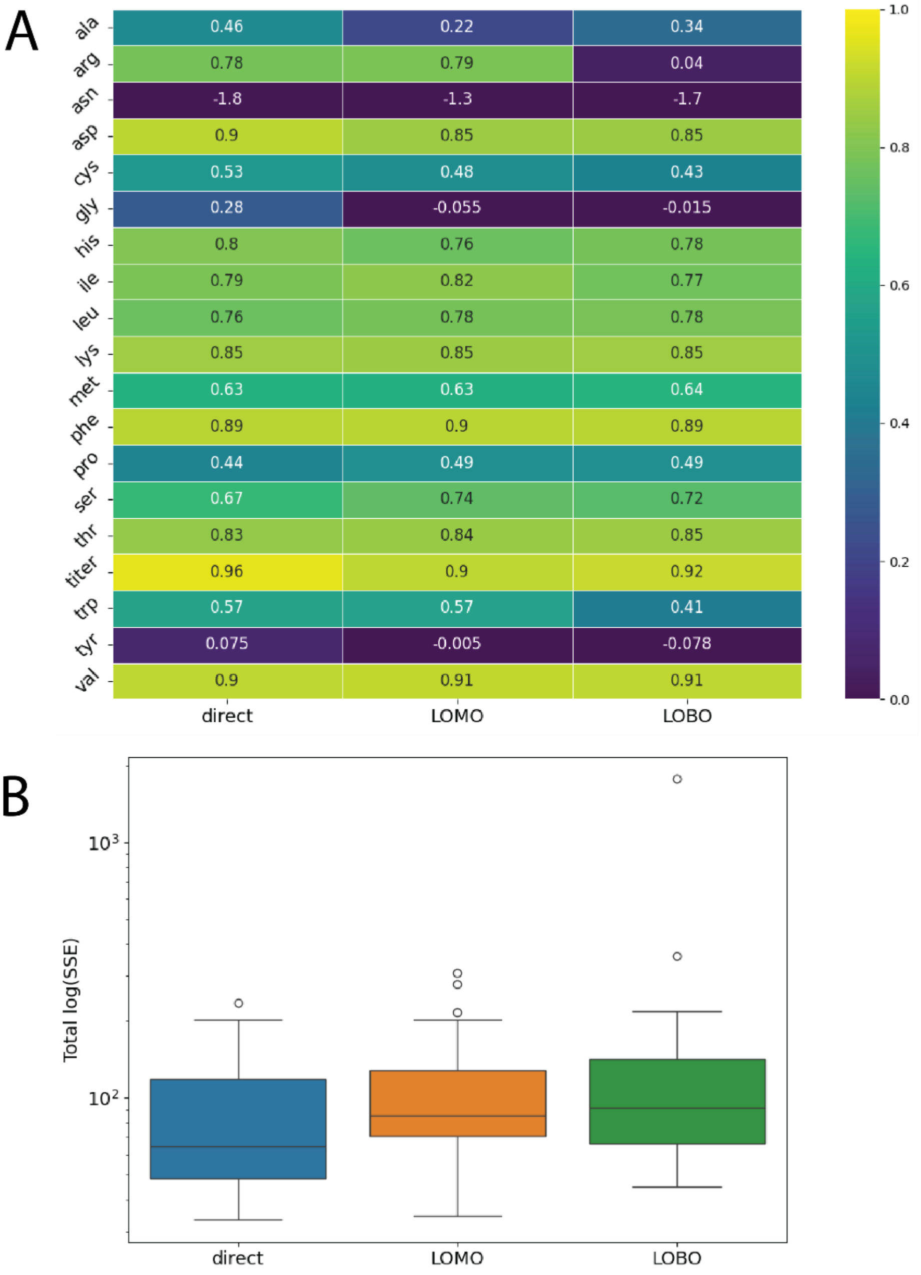

Supplementary Figure 8. Example of the propagation of the prediction variability of the PC-dFBA algorithm. Comparison of predicted (orange) and experimental (blue) time courses for viable-cell density, product titer, and 23 extracellular metabolites for batch CS4-5 under the LOMO_Flex configuration. The shaded blue regions represent MetRaC-derived confidence intervals. The shaded green regions represent FVA simulation envelope.

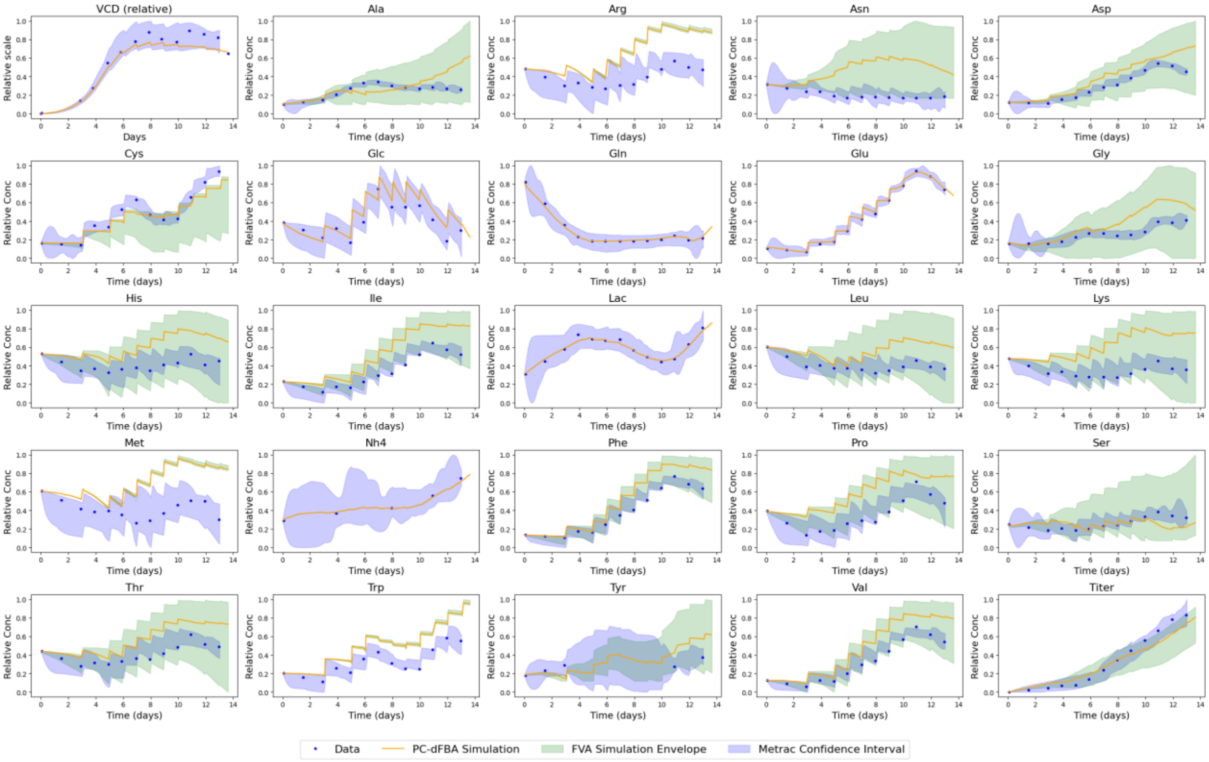

Supplementary Table 1. Summary of structural and functional changes in the iCHO1766 model during the five-step reduction process using MetRaC rates (95% confidence interval)

Model Reduction Steps	Reactions	Metabolites	Exchange Reactions	Genes	Essential Genes
Initial Model	6663	4455	602	1766	233
Step 1: Resolve Infeasibilities	4329	2231	284	1492	211
Step 2: MILP Exchanges	3563	1797	33	1366	195
Step 3: Transport	2164	1405	33	1134	185
Step 4: pFBA	575	468	32	595	138
Step 5: Thermodynamic Infeasible Loops	575	468	32	595	138

Supplementary Table 2. Summary of algorithm features evolution from FBA to PC-dFBA

Version	Key Feature	Temporal	Empirical Constraint Source
FBA	Mechanistic only	Pseudo steady-state	None
Hybrid FBA	Adds PCA-based constraints	Pseudo steady-state	PCA loadings
Hybrid dFBA	Dynamic extension	Time-discretized	PCA per interval
Hybrid dFBA - MOMA	Smooth transitions	Time-discretized	PCA per interval
PC-dFBA	ANN-predicted loadings to remove time dependency	Dynamic	NN regression (no interval definition)
